# Effectiveness of Using AI-Driven Hotspot Mapping for Active Case Finding of Tuberculosis in Southwestern Nigeria

**DOI:** 10.3390/tropicalmed9050099

**Published:** 2024-04-29

**Authors:** Abiola Alege, Sumbul Hashmi, Rupert Eneogu, Vincent Meurrens, Anne-Laure Budts, Michael Pedro, Olugbenga Daniel, Omokhoudu Idogho, Austin Ihesie, Matthys Gerhardus Potgieter, Obioma Chijioke Akaniro, Omosalewa Oyelaran, Mensah Olalekan Charles, Aderonke Agbaje

**Affiliations:** 1 Society for Family Health, 8, Port Harcourt Crescent, Area 11, Garki, Abuja 900247, Federal Capital Territory, Nigeria; aalege@sfhnigeria.org (A.A.); oidogho@sfhnigeria.org (O.I.); 2 EPCON, Schillerstr. 24, 2050 Antwerp, Belgium; sumbul@epcon.ai (S.H.); vincent@epcon.ai (V.M.); anne-laure@epcon.ai (A.-L.B.); 3 U.S. Agency for International Development, Plot 1075 Drive, Central Business District, Abuja 900103, Federal Capital Territory, Nigeria; reneogu@usaid.gov (R.E.); aihesie@usaid.gov (A.I.); ooyelaran@usaid.gov (O.O.); 4 Institute of Human Virology, Nigeria IHVN Towers, Emeritus Zone Plot 62, C00 Emeritus Umaru Shehu Ave, Cadastral, Abuja 900108, Federal Capital Territory, Nigeria; mpedro@ihvnigeria.org (M.P.); odaniel@ihvnigeria.org (O.D.); cmensah@ihvnigeria.org (M.O.C.); 5 EPCON SA, 11 Blombos Close, Sunnydale, Fish Hoek, Cape Town 7975, South Africa; thys@epcon.ai; 6 National Tuberculosis, Leprosy and Buruli Ulcer Control Programme, 16 Bissau St, Wuse, Abuja 904101, Federal Capital Territory, Nigeria; ocakaniro@gmail.com

**Keywords:** hotspots, tuberculosis, mapping, modelling, artificial intelligence

## Abstract

**Background**: Nigeria is among the top five countries that have the highest gap between people reported as diagnosed and estimated to have developed tuberculosis (TB). To bridge this gap, there is a need for innovative approaches to identify geographical areas at high risk of TB transmission and targeted active case finding (ACF) interventions. Leveraging community-level data together with granular sociodemographic contextual information can unmask local hotspots that could be otherwise missed. This work evaluated whether this approach helps to reach communities with higher numbers of undiagnosed TB. **Methodology**: A retrospective analysis of the data generated from an ACF intervention program in four southwestern states in Nigeria was conducted. Wards (the smallest administrative level in Nigeria) were further subdivided into smaller population clusters. ACF sites and their respective TB screening outputs were mapped to these population clusters. This data were then combined with open-source high-resolution contextual data to train a Bayesian inference model. The model predicted TB positivity rates on the community level (population cluster level), and these were visualised on a customised geoportal for use by the local teams to identify communities at high risk of TB transmission and plan ACF interventions. The TB positivity yield (proportion) observed at model-predicted hotspots was compared with the yield obtained at other sites identified based on aggregated notification data. **Results**: The yield in population clusters that were predicted to have high TB positivity rates by the model was at least 1.75 times higher (*p*-value < 0.001) than the yield in other locations in all four states. **Conclusions**: The community-level Bayesian predictive model has the potential to guide ACF implementers to high-TB-positivity areas for finding undiagnosed TB in the communities, thus improving the efficiency of interventions.

## 1. Introduction

According to the World Health Organization (WHO), around 10.6 million people across the world fell ill with tuberculosis (TB) in 2021. Until the COVID-19 pandemic, there was a yearly 2% decline in TB incidence being observed over the past 20 years, but the post-pandemic world actually saw an increase of 3.6% in the overall incidence of TB between 2020 and 2021 [1]. Between 2015 and 2021, the African region, where a quarter of all TB occurs, saw a decline of 22% in TB incidence, thus meeting the 2020 target of a 20% decline [1]. However, despite all efforts, more than 4 million, approximately 40%, of all incident cases remain undiagnosed or unreported to the National TB Control Program (NTP). Missing patients, especially the ones who go undiagnosed, can unknowingly transmit disease to their families and communities. Interventions need to be strengthened to find these missing TB cases and connect them to care.

Nigeria has the sixth highest burden of TB cases in the world, where 4.4% of all cases are found. The country is also among the top five countries that have the highest gap in estimated incidence and reported number of people with newly diagnosed TB [1]. In 2021, only 44% of the estimated number of incident cases were notified in the country. Finding the missing cases early and treating them remains the single most important priority in the country for TB control.

Apart from treating those with existing TB, it is imperative that the halting of transmission be prioritised. A disease like TB, which can remain asymptomatic for long periods of time, will need more than just passive case finding to control. Active case finding (ACF) is about proactively reaching out to the community, finding people who have undiagnosed infections, and connecting those people to care [2]. Current evidence supports that ACF interventions can help to reach undiagnosed individuals early and are beneficial in low-resource settings. A multi-faceted community-based TB case-finding intervention in two southern states of Nigeria observed that the intervention led to a 138% increase in case detection as compared to the expected notifications in the absence of any intervention [3]. 

In another study focused on potentially high transmission settings like urban slums, they found a 6.4% TB positivity among the target population, of which 65% were smear positive. They reported that the high transmission could be due to poor living conditions and overcrowding [4]. Another ACF project implemented in Ebonyi state in Nigeria combined house to house outreach with ACF activity to target attendees of a health facility in the general outpatient departments, ANC and MCH clinics, and people living with HIV (PLHIV) reporting to ART centres—including contact tracing of index clients. They found 3.2% TB positivity among those who were evaluated [5]. Facility-based screening like this can reach a big population in a relatively short period of time, but the overall impact can be variable. Although ACF interventions are necessary and impactful, they can be resource intensive. Innovative and data-driven approaches can make them more efficient and help to reduce the overall resources consumed. An early warning outbreak recognition system (EWORS) was implemented in 14 states in Nigeria, which helped to identify areas with potentially high TB spread at the ward level, and subsequent ACF interventions in the hotspot wards yielded a significantly lower number needed to screen as compared to non-hotspot wards [6].

It is now advocated that communities use locally tailored interventions to target specific areas that could be at increased risk of TB. Subnational estimation of TB burden, though extremely valuable to guide local community-based interventions, is rather limited in low–middle-income countries. Most countries still depend on case notifications, prevalence surveys with small-area estimation, and surveys of infection to estimate burden of TB. Although such surveys are expensive and time consuming, case notifications are often subject to bias due to differential access to healthcare and under-reporting in low- and middle-income countries [7]. The MATCH framework proposed by Rood E et al. emphasises using subnational data such as disaggregated notifications and local screening data to derive granular insights on a subnational scale [8]. Also, the risk of TB transmission and delayed diagnosis and treatment is determined by several contextual factors like population demographics, socioeconomic conditions, nutritional status, access to health services, and environmental conditions, which are important to consider along with notification data [9,10,11]. Therefore, there is value in leveraging data and available technology to make evidence-based decisions for routine programmatic activities and improve the effectiveness of interventions [12]. 

The goal of this paper is to describe the approach taken for developing a TB risk predictive model at the community level and discuss the impact it had on the yield of TB ACF interventions. The ACF yield in clusters that overlap with model predicted hotspot locations was compared with those that do not overlap with hotspots, our hypothesis being that if implementers selected sites that had high predicted TB positivity rates for ACF—“TB hotspots”—they would find more cases of undiagnosed TB than at sites not predicted to be hotspots.

## 2. Methodology

**Data generation methodology.** A retrospective analysis was conducted of the data generated from community-based ACF interventions led by the Society for Family Health (SFH) in Nigeria in four southwestern states, namely, Lagos, Oyo, Ogun, and Osun. The project was funded by the United States Agency for International Development (USAID), which established the Local Organizations Networks (LON) to increase the level of TB cases detected and treated in Nigeria over five years (2020–2025). The project engages community-based organizations (CBOs) and community volunteers (CVs) for contact tracing of index TB cases enrolled for treatment, community outreach, active case finding, and sensitisation meetings in the communities. 

**Implementing a closed-loop dynamic model training pipeline.** Principles of machine learning were used to develop an epidemiological digital representation (“twin model”) of the TB situation in the four states of Nigeria. This digital twin model was based on data generated from local ACF program implementation and contextual data and followed the principles of the MATCH framework as implemented in Pakistan TB ACF settings [8,13,14]. The outputs generated by the model were visualised on a web interface (hereafter referred to as the geoportal) that was used by the local teams to identify high-priority neighbourhoods for routine activities. The new data thus generated from the routine ACF events were regularly incorporated into the training data. Continuous flow of new data from the program formed a feedback loop, improved the model’s ability to learn from data coming in, distinguished better among the low- and high-TB-risk areas, and calibrated previous predictions. This method allowed the outputs to become fine-tuned over time, keeping in mind the need to support program teams in finding the last missing TB cases. The first model was trained in September 2020 with very limited data, including only 24 unique ACF locations. This grew to the present 857 unique locations as of June 2022. 

**Justification for a Bayesian modelling approach.** The approach used Bayesian networks that provided a powerful machine learning technology to reason with uncertainty in complex environments. In this case, it refers to the spatially aware programmatic and socioeconomic data, multiple variables with potentially nonlinear relationships, large amounts of natural variation, and missing values. The model was queried for TB positivity rate across the four states, including areas where observations were not available, using known local contextual data at the relevant subnational resolution. The Bayesian framework used was a proprietary naive Bayes implementation. Naive Bayes is founded on the assumption of conditional independence between predictors—called the naive Bayes assumption. The benefits of such an approach are well known in terms of ease of implementation, scalability to the number of predictors and data points, and ability to be trained on relatively limited input data. 

**Data geolocation.** Routinely collected TB screening data from ACF sites were received in monthly intervals. Each row corresponded to a unique location or community where the screening activity took place and contained corresponding information on the state, local government area (LGA), and ward name. Google Maps was used to manually look up the geo-coordinates of each community, and the ones that did not show up on Google Maps were excluded. 

**Data preparation and transformation.** The TB positivity rate (proportion) was derived from the number of people diagnosed with bacteriologically positive TB and absolute number of people screened at each ACF event. Similar proportions were also calculated for contact investigations (close contacts of index TB clients diagnosed positive over total contacts screened) and facility-based screening (number of attendees diagnosed positive over number of attendees screened). 

**Socioeconomic data processing.** Indicators of sociodemographic situation and human development known to be associated with TB [10,15] were accessed from open-source platforms. Data on age- and gender-related population estimates [16], population density [17], poverty [18], nighttime lights [17], and elevation [17] were obtained from WorldPop. Spatially modelled data from the Demographic and Health Surveys (DHS) platform on literacy, access to clean water, sanitation services, stunting in children (proxy for nutritional status), vaccination coverage (indicator of access to care and health-seeking behaviour) were used [19]. Travel time to health care facilities [20], distance to major roads [17], and health facility density [21] were also used as indicators of access to care. Modelled estimates of human immunodeficiency virus (HIV) prevalence [22] and child mortality [23] were available from the Institute for Health Metrics and Evaluation (IHME) and the Global Health Data Exchange (GHDx) platform. A more detailed view on the variables used is provided in Table 1. 

**Population clustering and Thiessen polygon generation.** In highly populous countries like Nigeria, the usual administrative units like the wards can be very large in some regions. In order to provide a model output that is able to guide neighbourhood-level interventions, wards were further divided into smaller units. This was done by an observation-weighted k-means clustering [24,25], algorithm that divided the population in a given ward into polygons of varying shape and population, such that each polygon contained approximately 10,000 people and did not cross the ward borders. This way, the four states were disaggregated into 7088 population clusters (also called Thiessen polygons) [26] of variable sizes. High-population-density areas had smaller size clusters, whereas low-density areas like rural areas had comparatively large-sized clusters. K-means clustering is a well-known unsupervised machine learning technique for finding clusters in data. The benefits of using an unsupervised algorithm to cluster population density include estimates of local population density centroids (from the k-means cluster centroids), facilitating microplanning in the absence of high-resolution municipal boundaries. The clusters also allowed our clients to set targets based on the proportion of each Thiessen population to be screened. The model was trained to produce an output for each of these 7088 population clusters. 

**Data aggregation and standardization.** Each variable mentioned in Table 1 was aggregated to match the resolution of the newly designed polygon level and scaled to a rate form, such that each population cluster had a unique profile defined by its local contextual information. 

**Model training and hotspot recommendations.** The model was trained on the TB positivity rate derived from ACF events that took place in a limited number of population clusters to predict a TB positivity rate for all other clusters. The predicted output thus allowed for identification of other clusters that could be prioritised for ACF activities. The geoportal allowed the local teams to activate a filter and select sites with the highest predicted TB positivity rate in their region of activity. The location was then communicated to the field teams responsible for organising TB screening events in the community. The yield obtained at the ACF event (observed TB positivity rate) was allocated to the respective population cluster to calculate the new average observed rate. If the site had never been screened, the new yield obtained after ACF was included as a new data point on the dataset. The model was dynamic in nature, receiving new ACF data at regular intervals and retrained every two to three months. The outputs were expected to improve over time, and the model identified hotspots or population clusters that could be at risk of increased TB positivity driven by the local contextual conditions such as poverty, access to health facilities, or population density. 

**Platform-assisted program steering.** The hotspot predictions had been used in the four states since early 2021 for selecting the most optimal sites for ACF; however, the uptake was variable across the region. Some of the ACF events were planned using the model predictions, whereas others were selected based on the conventional approach. The latter was based on facility-level TB notifications, aggregated to the LGA level, which serves as the functional unit of the TB control program in Nigeria. LGA-level data were submitted to the State TB and Leprosy Control Officer (STBLCO), who then reported to the NTP [27]. Thus, facility-level notifications were used to identify the catchment areas with potentially high TB burden, which were then targeted for active case finding. The project implementers who preferred to select their ACF sites using the geoportal commonly chose sites with high predicted TB positivity rates, from the top 10% to the top 50%. 

**Comparing hotspots and non-hotspots.** The ACF yield in population clusters that overlap with model-predicted hotspot locations was compared with those that were not predicted as hotspots (other sites). These other sites were chosen based on the conventional approach of using facility-level notifications. The comparison was carried out between proportions of TB positivity (yield) using the Chi-square test. For the sake of uniformity, the threshold was set at 30%—all clusters that had a TB positivity rate falling in the top 30% range of predicted values in each state were classified as hotspots. 

**Investigating the relationship between covariates and model output.** Pearson’s correlation analysis was performed for all covariates and the output variables in a pairwise manner to investigate their magnitude and the direction of the relationships as a data-quality check and to investigate the impact of various covariates on the model outputs. Significant relationships between covariates and the output variable could provide clues to causal or confounding relationships in the data warranting investigation.

## 3. Results

**Figure 1:**
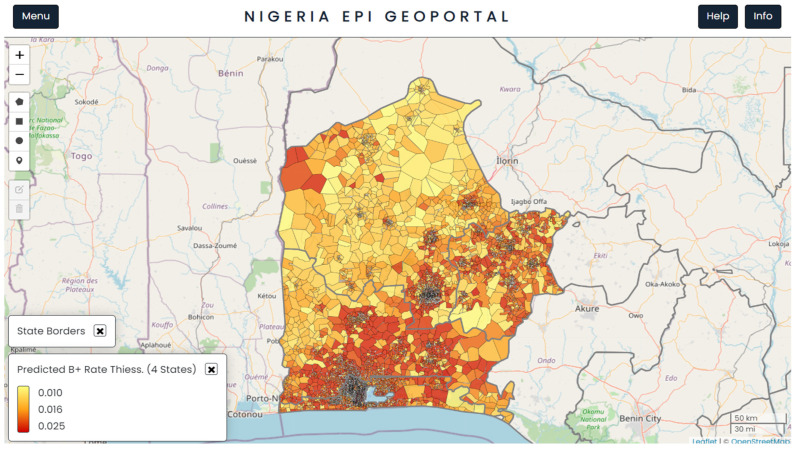
A visualisation from the TBLON 3 geoportal showing the four states subdivided into population clusters and the spatial distribution of the predicted bacteriological TB positivity rate. The map is in north-up orientation.

The TB positivity rate was predicted at the population cluster level (below ward level) in four southwestern states of Nigeria (Figure 1). The predicted outputs were used to select more suitable ACF sites, a step towards making data-driven decisions. To facilitate reporting the predicted TB burden at standard municipal boundaries, population-weighted averages of the predicted rates at the cluster level were also calculated at the ward level, which is the lowest municipal level in Nigeria (see Supplementary Data File S1).

**Figure 2:**
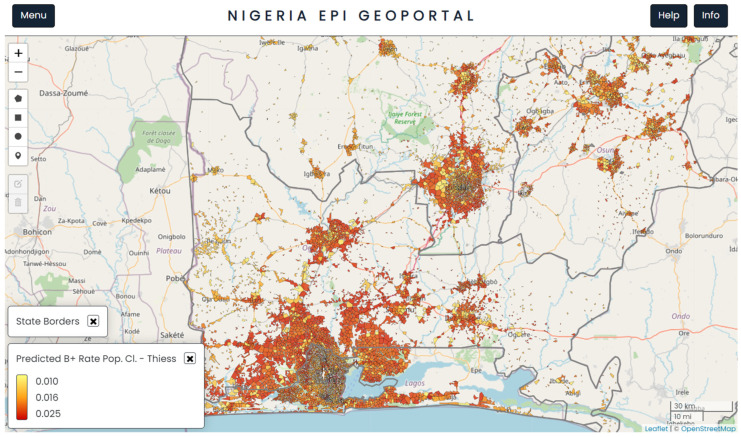
A visualisation from the TBLON 3 geoportal showing the distribution of human settlements across the four states (within the population clusters) and the spatial distribution of the predicted bacteriological TB positivity rate. The map is in north-up orientation.

Figure 2 shows a further refined view of Figure 1, limited only to the areas that were inhabited by individuals after eliminating all unsettled areas such as fields, forests, and barren land. The areas with clustering of a large number of settlements represent major cities and urban areas, surrounded by less densely packed settlements in peri-urban and rural areas. 

Table 2 describes the overall administrative division, the number of population clusters further created during this modelling exercise, and the total number of ACF locations that could be mapped using the approach described earlier. In the four states combined, the mappable population clusters formed 12% of the total 7088 clusters, and the respective data generated were used for model training.

**Figure 3:**
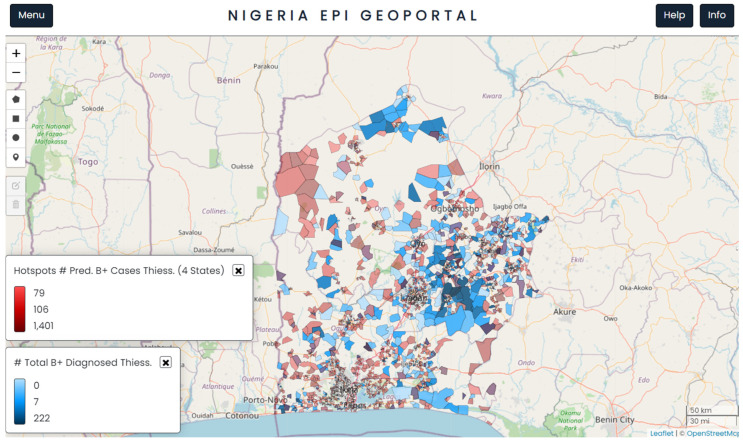
A visualisation from the geoportal showing the spatial distribution of predicted bacteriological TB positivity rate in red and previous ACF activity in blue, at population cluster level. The map is in north-up orientation.

Figure 3 shows the geographical distribution of ACF sites mapped across the four states (in blue) together with the population clusters identified as hotspots (in red) by the model. The geoportal allowed the user to visualise several layers of interest, such as previous ACF activity, locations of TB diagnostic services, and newly predicted hotspots. 

**Figure 4:**
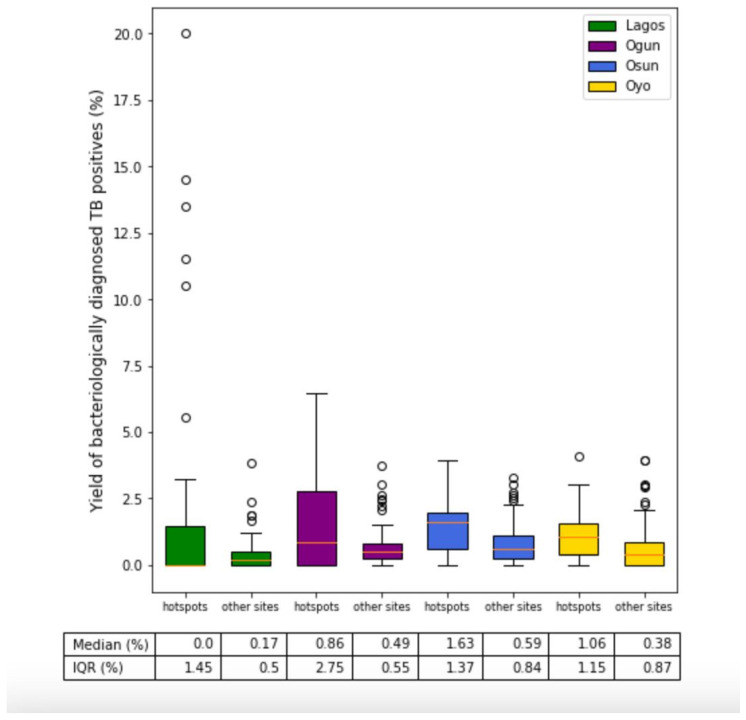
Graphical comparison of median TB positivity yield obtained when ACF activity took place in the predicted hotspots in comparison with other sites for each of the states. The red lines indicate the median values, and the whiskers extend to 1.5 times the interquartile range above and below the third and first quartiles, respectively. Circles are individual data points plotted as outliers.

Figure 4 shows the graphical representation of the median TB positivity yield obtained when ACF activity took place in the predicted hotspots in comparison with other sites for each of the states. The median yield in hotspot clusters was higher than the non-hotspot clusters in three out of four states. The lowest median yield was observed in Lagos in the hotspot locations, albeit with exceptionally high yield in some locations; thus, the average yield was still higher than the non-hotspot group for the state as a whole. Outliers seemed to be common in all states and in both groups. 

Table 3 shows the comparative analysis of average ACF yields obtained in each of the states. For each state, the table depicts the outcome of ACF in the predicted hotspots, sites that were identified by the model to be at high risk of TB, and other sites that were not predicted to be among high-TB-positivity locations but were nevertheless chosen for ACF by the local teams based on the conventional approach.

**Comparison of yield differences between hotspot and non-hotspot sites.** In terms of ACF coverage of the predicted hotspots within each state, Lagos had the highest coverage at 8%, while Ogun state only selected 3% of its predicted hotspots for screening. Fewer sites were selected based on the model predictions (128), and the conventional approach (729) seemed to be more popular overall in the four states. Even though much less screening happened in the predicted hotspots, the proportion of TB-positive individuals diagnosed was at least 1.7 times higher individually in each state, and the difference was statistically significant. In Osun state, the yield obtained in the predicted hotspots was more than double compared to that in the other sites. Although Ogun state had the lowest uptake of predictive sites for ACF, they reported the second highest overall yield, which was 95% more than that observed in the other sites selected conventionally. 

**Investigating the relationships between model covariates and model predictions.** Based on Pearson’s correlation between covariates and the predicted output variables, the variable with the highest correlation with the bacteriologically positive predictions was “evaluated_tot_norm_f” (observed evaluated rate at the facility level, the number tested divided by total screened), with a positive correlation (r = 0.73, *p*-value = 0). Similarly, “presumptives_tot_norm_f” (presumptive rate at the facility level) also correlated highly with predicted B-positive rates (r = 0.719, *p*-value = 0), while all-form diagnosis and B-positive rates were slightly less correlated with predicted B-positive rates (r = 0.413, *p*-value = 4.801 × 10^−154^ and r = 0.388, *p*-value = 6.4685 × 10^−135^, respectively). Conversely, the variable with the lowest correlation to the predicted bacteriologically positive rate was the total screened at the facility level (r = −0.335, *p*-value 1.79 × 10^−98^). The contextual variable with the highest correlation to predicted B-positive rates was HIV prevalence (r = 0.12, *p*-value = 0) and the least correlated contextual variable was population density of people over 60 years old (r = −0.133, *p*-value = 0). See Supplementary Data File S2.

## 4. Discussion

**Using community ACF data for high-resolution TB burden modelling.** Routine ACF data generated at the community level were used to make a high-resolution TB predictive model for the four southwestern states in Nigeria. The outputs were accessible to the local team via a customised geoportal, which allowed the ground teams to visualise the hotspots on a mapping portal and make data-driven decisions for their ACF activities. Apart from planning community-based TB screening, the predictive outputs were also used for other program objectives, such as to identify priority areas for engaging private care providers and community engagements. 

**Using unsupervised population clusters combined with local context to model underserved locations.** This predictive model is the first of its kind in Nigeria to predict hotspots on specially designed population clusters below the ward level. These population clusters were designed in discussion with the local teams such that each cluster contained a manageable size of population (up to 10,000) for ACF. Identifying the high-TB-transmission areas on the basis of facility-level notification data alone can be challenging. This model does not depend heavily on the number of registered TB clients at the facility level, which is often affected by the capacity and quality of diagnostic and treatment services available in an area. Areas that are rather remote and located away from quality TB diagnostic facilities can have low notification rates and might be mistaken for low TB transmission [7,15,28].

In contrast, a study in Kampala, Uganda, reported that facility-based passive case finding could predict high-TB-prevalence settings and was sufficient to effectively guide ACF. However, their study area was a 2.2 km^2^ region with a population of approximately 49,000 individuals with quite homogenous access to TB diagnostic and treatment services. The authors remarked that facility-based notifications may be less useful in bigger regions where access to health services is variable [29]. Therefore, it is plausible that the current approach is well suited for these populous southwestern states in Nigeria. 

Leveraging limited ACF data with local context for data-driven program steering. This model leveraged community-based screening data from a limited number of locations, enriched it with other local determinants of TB transmission, and predicted the TB positivity rate at the community level across the whole geographic region. The advantage of using ACF data could be that it reflected the TB transmission among the people who live or frequent the screened location. The model thus predicted and helped to identify even those communities that were traditionally not known to report many TB cases or were not reached by any previous ACF activities. This can also be seen as an added advantage over the commonly used spatial clustering techniques. These techniques are able to identify statistically significant clustering patterns from heterogenous disease distributions. Although they would detect significant clusters if several data points fell adjacent to each other, they can fail to identify small areas of TB transmission if they exist in an isolated and remote location [30]. Identification of disease hotspots at the local level is believed to be one of the important components of epidemic elimination [7]. Thus, the local teams could make informed decisions regarding selecting ACF sites and reach out to the communities to find a higher proportion of undiagnosed TB. 

**Population density associated with higher predicted TB rates.** It can be seen that the highest-TB-risk areas were predicted to be in and around the densely populated urban centres. Although urban centres are often characterised by high accessibility to better health care services, they also typically have overcrowded houses and people living in lower socioeconomic conditions, which are determinants of TB transmission [31,32]. 

**Increased yield from model recommendations obtained.** TB positivity yield obtained at the ACF sites that were also predicted by the model to be at high risk of TB were compared to other sites that were selected based on the conventional approach for ACF planning (using notification data). The overall yield in predicted hotspots was 73% higher in Lagos, 95% higher in Ogun, 103% higher in Osun, and 75% higher in Oyo state compared to the other sites, and the results were statistically significant. The results were especially impressive because the model-recommended ACF sites only formed a very small proportion of the total ACF that took place across the four states. This supports the fact these model-predicted hotspots were in fact better suited for ACF, whereas the non-hotspot sites did not find as many new TB cases with extensive screening. Ogbudebe et al., who used their EWORS system to identify TB hotspots in 14 states of Nigeria, reported that the number needed to screen to diagnose a TB case in the hotspot and non–hotspot areas was 146 and 193 per 10,000 people, respectively, which translated to an almost 24% higher yield [6]. 

**Potential and challenges for targeted ACF in highly populated cities.** Looking at Lagos state, the median yield observed in the predicted hotspots was almost zero, which means up to 50% of ACF events did not find any TB cases. Although the overall yield (average) was higher as compared to the other sites, it potentially could have been driven by the locations that found an extremely high yield. Lagos is the most densely populated state in Nigeria, with a population density of almost 3791 per square kilometre. The state is also the centre of focus for the National TB Control Program, with several government and non-government organisations implementing their TB case-finding intervention programs across the state. It is possible that some population clusters were repeatedly screened by separate organisations at different points of time over the past few years. This could have been one of the reasons for it having the lowest median yield among the four states despite having the highest extent of screening. This also points towards a need for increasing collaboration among locally active organisations that perform ACF and sharing of data to make evidence-based decisions. Pooling of data from different ACF programs across the state could further boost predictive models like these and benefit the whole community. A recent study from Pakistan that retrospectively analysed their ACF program data found that among more than 1500 individual ACF events conducted in and around Karachi city, almost three fourths did not find any TB cases. However, a small proportion of 5% of events accounted for 40% of the TB cases diagnosed. The authors concluded that a more targeted approach for ACF in high-population metropolitan cities can increase the yield and cost-effectiveness of interventions [33].

**The benefits of using local context and ACF data for TB burden modelling.** The interquartile range of yield, as observed in sites other than hotspots, reflects that the sites selected by the local teams could have had similar local context and TB burden. It is common practice that in most states ACF sites are chosen based on facility-level notifications. However, the broader range and extreme values of yield found in predicted hotspot locations show that even with much fewer sites, these were potentially different from those selected by the conventional approach (non-hotspot sites) and had a much higher number of undiagnosed TB. The reason could be that the predictive model learnt from local contextual and ACF data to uncover TB transmission sites, which could have been easily missed if only aggregated notification data were used. Further, training models with local context could potentially ameliorate the effects of underreporting or other systematic reasons for low notification rates, which may not accurately reflect the true disease burden on their own.

**Limitations of only using notification data for program steering.** Another reason for these high yields could be that the predicted hotspots were previously seldom targeted based on reluctance of the local teams to explore communities that are not justifiably represented on the treatment registers (facility notification registers). This leaves a pool of undiagnosed TB waiting to be diagnosed at the earliest opportunity.

**Local TB burden heterogeneity necessitates local approaches to ACF.** Similarly, a study in Peru mapped geographic coordinates of individuals treated for TB in an urban district over five years and analysed their spatial distributions. They reported heterogeneous distributions of TB, with clustering of local hotspots and cold spots across the 74 neighbourhoods in the district. The study found that although the median rate of reported cases in the district was 123.6, the range among neighbourhoods varied from 0 to 800 cases per 100,000 members of the population. Although the study utilised notification registers alone, it highlights the possibility of local epidemics and the relevance of geographic mapping of individual cases from their addresses to identify areas at risk of TB transmission and its practical implications for decision-making [34]. 

**Challenges to the implementation and uptake of model-assisted ACF.** There are a number of challenges associated with deployment of the geoportal and model-driven case finding. It took some handholding and mentorship before the local teams could trust the predictions, as a number of the predicted hotspots were not known to be high yielding in the past. Also, the limited digital literacy of some of the local teams particularly restricted the use to some cadres of field staff. Additionally, it was challenging to input data back into the model, as reporting was basically paper based, with sparse electronic data capturing. 

**Using a predictive model allows for the identification of new ACF sites.** The main strength of this approach was that the local ACF data allowed predictive models to learn about the distribution of undiagnosed TB cases in the community, thus improving their potential to predict new ACF sites, as opposed to the facility-level notification data. This approach leverages incremental learning and does not require large volumes of data from the beginning, allowing for data-driven decision-making in low-resource settings.

**Limitations of the approach.** Our results should be interpreted in light of certain limitations and assumptions. Individuals screened at a certain population clusters or communities were assumed to belong to that location; hence, the routine data collection sheets reported aggregated and not individual-level data. The geo-coordinates of the ACF sites were manually searched for online and mapped; this could have led to some discrepancies in precise mapping. Also, it was not possible to find all communities on Google Maps, and thus, they had to be excluded. The uptake of using models for ACF planning was variable across the states, and we had no control over how frequently this approach was being used or the way this approach was being used. The retrospective nature of this analysis did not allow us to control for any biases that could have had an impact on the way the screening events were planned. For example, if the community volunteers knew that they were screening in a predicted hotspot, some of their practices could have been affected. Also, because the total training data at the population cluster level were very limited compared to the total number of clusters the model was predicting for, it was not possible to perform further disaggregation into age groups or gender, or train the model on unseen data. The current work was unable to show the overall impact of this case-finding intervention on the total notifications in the four states, as the TB-LON 3 program did not have access to all the notification data at the state level. Nevertheless, notifications to the National TB Control Program are affected by a number of factors, including the diagnostic and treatment initiation capacities, whereas the model predictions were only used to guide the ACF site selection. Finally, as the analysis was carried out retrospectively, hotspots predicted from the final training set may not have been predicted as hotspots at the time of screening and could have been selected by the conventional approach; thus, prospective studies are needed to more accurately quantify the improvement in yield of the data-driven approach when used for targeted ACF. Potential confounding factors identified by Pearson’s correlation analysis included a negative relationship between total number of people screened at the facility level and the predicted B-positive rates. Since facilities that are in communities with better health infrastructure may tend to screen more patients, this would create an artificial relationship between the total number screened and B-positivity, although it can be argued that the total number screened does provide indirect evidence for access to care in a community. Overall, the relationships identified between Pearson’s correlation between covariates and predicted B-positivity were in line with the expected relationships in the field, such as the positive correlation between HIV prevalence and predicted B-positivity by the model.

**Future directions for locally targeted ACF.** Based on the findings of this work, for future ACF programs and interventions, the recording of geographic information such as coordinates should be promoted as much as possible. These results are motivating enough to increase confidence in such data-driven approaches. A more systematic and large-scale comparison in a prospective intervention design can provide stronger evidence for advocating for this approach in other regions.

## 5. Conclusions

This approach leveraged local data, looked beyond facility-level aggregated notification, and enabled outreach to previously underserved locations. The program implementers were able to find much higher yields when ACF activity happened in the model-predicted hotspots.

## Figures and Tables

**Table 1:** Description and sources of all variables used for training the predictive Bayesian inference model.

Variable Name	Description/Definition	Source	Resolution	Year
Total population density	Number of people per square kilometre	WorldPop	100 m	2020
Male population density	Counts of males per square kilometre	WorldPop	100 m	2020
Elderly population density	Counts of people age 65 plus per square kilometre	WorldPop	100 m	2020
Population growth model	Modelled estimates using 2000, 2010, 2020 populations	WorldPop	100 m	2010–2020
Poverty index	Proportion of people below the poverty line of USD 1.25 a day per 1 × 1 km grid cell	WorldPop	1 km	2013
Access to improved water source	Percentage of the de jure population living in households whose main source of drinking water is an improved source	DHS	5 × 5 km	2018
Access to improved sanitation facilities	Percentage of the de jure population living in households whose main type of toilet facility is no facility (open defecation)	DHS	5 × 5 km	2018
Prevalence of stunting in children	Percentage of children stunted (below −2 SD of height for age according to the WHO standard)	DHS	5 × 5 km	2018
Vaccination coverage (8 basic vaccinations, DPT1, DPT3, measles)	Percentage of children 12–23 months old who are vaccinated	DHS	5 × 5 km	2018
Literacy (men, women)	Percentage of men and women who are literate	DHS	5 × 5 km	2018
Motorised travel time to healthcare facility	Optimal travel time to healthcare with access to motorised transport	Malaria Atlas Project	1 × 1 km	2019
Distance to major roads	Distance of a major road from the centroid of a population cluster, measured in metres	WorldPop	100 m	2016
Child mortality under age 5	Estimates of death counts for children under the age of 5 (0–5 years old)	IHME	5 × 5 km	2017
HIV prevalence	Estimated prevalence among individuals aged 15–59 years	IHME	5 × 5 km	2017
Health facility coverage (density)	Number of health facilities per square kilometre	GHDx	point level data	
Nighttime lights	VIIRS data measured in nanoWatts/cm^2^/sr	WorldPop	100 m	2016
Elevation	Elevation above sea level (in metres)	WorldPop	100 m	2016
TB Program Variables				
Active case-finding yield	Number of people diagnosed with TB (all forms of TB and bacteriologically positive) over absolute number of people screened	TBLON program	Community level	
Intensive case-finding yield	Number of attendees diagnosed positive over number of attendees screened			
Contact investigation TB positivity yield	Close contacts of index TB clients diagnosed positive over total contacts screened			

**Table 2:** Administrative division and population distribution across the four states.

Characteristics of the Administrative Unit	Lagos	Ogun	Osun	Oyo	Total of Four States
Population of the state	12,594,007	6,375,060	4,871,838	8,308,362	32,149,267
Number of LGAs	20	20	30	33	103
Number of wards	377	226	267	344	1214
Total number of Thiessens	2706	1403	1120	1859	7088
Number of Thiessens with any ACF activity mapped	271	132	173	281	857
ACF coverage (%)	10.01%	9.41%	15.45%	15.12%	12.09%

**Table 3:** Comparative analysis of average ACF yields obtained in predicted hotspots and other sites separately for each of the states.

Characteristics of ACF per State	Lagos		Ogun		Osun		Oyo		Total of Four States	
	Predicted Hotspots	Other Sites	Predicted Hotspots	Other Sites	Predicted Hotspots	Other Sites	Predicted Hotspots	Other Sites	Predicted Hotspots	Other Sites
Total number of Thiessens in the state	811	1895	420	983	336	784	557	1302	2124	4964
Thiessens selected for ACF	67	204	12	120	22	151	27	254	128	729
Screening coverage %	8	11	3	12	7	19	5	20	6	15
Number of people bacteriologically diagnosed TB positive (P)	132	917	26	716	371	2325	144	1470	673	5428
Number of individuals screened (S)	20,075	243,516	2047	109,675	18,863	240,692	12,069	216,929	53,054	810,812
Yield % [(P/S)×100]	0.66	0.38	1.27	0.65	1.97	0.97	1.19	0.68	1.27	0.67
*p* value	**<0.001**		**<0.001**		**<0.001**		**<0.001**		**<0.001**	
% Difference in Yield	73.68		95.38		103.09		75		89.55	

## Data Availability

The data that support the findings of this study are available from the Institute of Human Virology, but restrictions apply to the availability of these data, which were used under licence for the current study and so are not publicly available. Data are, however, available from the authors upon reasonable request and with the permission of the Institute of Human Virology.

## Supplementary Materials

The following supporting information can be downloaded at: https://www.mdpi.com/article/10.3390/tropicalmed9050099/s1, File S1: Ward level TB risk predictions; File S2: Pearson’s correlation analysis between covariates and model outputs.

## Abbreviations

ACF	Active case finding
CBO	Community-based organization
CV	Community volunteers
DHS	Demographic and health surveys
EWORS	Early Warning Outbreak Recognition System
GHDx	Global health data exchange
HIV	Human immunodeficiency virus
IHME	Institute for Health Metrics and Evaluation
LGA	Local government area
LON	Local organizations network
NTP	National Tuberculosis Control Program
SFH	Society for Family Health
STBLCO	State Tuberculosis and Leprosy Control Officer
TB	Tuberculosis
USAID	United States Agency for International Development
WHO	World Health Organization
